# Discoveries from ornamental jellyfish in aquaria—description of *Malagazzia michelin* sp. nov. (Cnidaria, Hydrozoa, Leptothecata), second species of the genus from Japan

**DOI:** 10.3897/zookeys.1268.173354

**Published:** 2026-02-02

**Authors:** Takato Izumi, Shuhei Ikeda, Yuichi Nozoe, Sota Goto, Natsumi Imamura, Tadaaki Kinoshita, Hiroaki Uchida, Yoshimi Hamatsu, Hisashi Akiyama, Kazuya Okuizumi

**Affiliations:** 1 Department of Marine Bio-Science, Faculty of Life Science and Biotechnology, Fukuyama University, 985 Sanzo, Higashi-Mura Cho, Fukuyama-City, Hiroshima 729-0292, Japan Fukuyama University Fukuyama Japan; 2 Tsuruoka City Kamo Aquarium, 657-1 Okubo Imaizumi, Tsuruoka, Yamagata Prefecture 997-1206, Japan Tsuruoka City Kamo Aquarium Tsuruoka Japan; 3 Saikai National Park Kuju-kushima Aquarium, 1055 Kashimae, Sasebo, Nagasaki 858-0922, Japan Saikai National Park Kuju-kushima Aquarium Sasebo Japan; 4 Suo-Oshima Town Nagisa Aquarium, 2211-3 Ihota, Suo-Oshima, Yamaguchi 742-2601, Japan Suo-Oshima Town Nagisa Aquarium Suo-Oshima Japan

**Keywords:** Exhibition, hydrozoan, Leptomedusae, lifecycle, Malagazziidae, Nagasaki, Suo-Oshima Island, taxonomic key

## Abstract

*Malagazzia michelin***sp. nov**. is described as the ninth species in this genus. Medusae were collected in shallow waters of western Japan and observed in several aquaria, and cultures were maintained at the Tsuruoka City Kamo Aquarium and Saikai National Park Kuju-kushima Aquarium. Adult medusae were identified as belonging to the genus *Malagazzia* owing to the presence of excretory papillae on the tentacular bulbs and rudimentary bulbs, four gonads surrounding radial canals, and manubrium with four lips. The obtained polyps also exhibited the typical characters of this genus. However, these medusae can be distinguished from those of other species of *Malagazzia* by noticeable brown spot-like structures on the gonads and manubrium; combinations of several characters also suggest that the medusae represent an undescribed species. *Malagazzia michelin***sp. nov**. is also demonstrated to be a *Malagazzia* species by a phylogenetic analysis using DNA sequences. Thus, we determine these individuals as the second and a new species from Japanese waters. We also discuss the challenge of common names used for medusae in aquaria in Japan.

## Introduction

Malagazziidae Bouillon, 1984 (Hydrozoa, Leptothecata) is a family of jellyfish in the class Hydrozoa. This family includes three genera: *Malagazzia* Bouillon, 1984; *Octophialucium* Kramp, 1955; and *Tetracanna* Goy, 1979 (Bouillon et al. 2006; Schuchert 2024). Another previously included genus, *Octocanna* Haeckel, 1879, is considered to be nomen dubium (Schuchert 2024). The type genus, *Malagazzia*, contains eight nominal species, none of which have been currently designated as type species for the genus. Most recently, *Malagazzia hirsutissima* Akiyama, Horinouchi & Kubota, 2013 was described in Japan. In Malagazziidae, *Malagazzia* is characterized by four radial canals completely surrounded by linear to ribbon-like gonads, adaxial excretory papillae on each tentacular bulb and marginal wart, closed statocysts, manubrium with four lips, and no gastric peduncle (Bouillon et al. 2006; Akiyama et al. 2013).

However, we should pay attention to the detail of marginal warts and excretory papillae: in Malagazziidae, it is suspected that no permanent marginal warts and every wart probably grow tentacular bulbs (= rudimentary bulbs; Wang et al. 2018). Both *Malagazzia* and Malagazziidae were established by Bouillon (1984) by the division of the genus *Phialucium* Maas, 1905. The key morphologies of this taxonomic operation are marginal warts (sometimes written as rudimentary bulbs) and excretory papillae (Bouillon 1984). *Phialucium mbengha* (Agassiz & Mayer, 1899), the only species remaining in the family Phialuciidae Kramp, 1955, has permanent rudimentary bulbs. Kramp (1961) mentioned that Phialuciidae has normally four radial canals, with permanently rudimentary tentacle bulbs and marginal bulbs with adaxial excretory papillae. However, Bouillon (1984) showed that *P. mbengha* does not have excretory papillae and these structures are not apparent in the illustrations by Agassiz and Mayer (1899) and in the subsequent studies. Therefore, Malagazziidae and Phialuciidae can be distinguished by the presence of excretory papillae. Bouillon et al. (2004) mentioned that rudimentary bulbs are not permanent but generally develop into tentacles, so in *Malagazzia* their number decreases with the size of the jellyfish, in contrast to *Phialucium* of which rudimentary bulbs never change to tentacular bulbs. Also, Wang et al. (2018) stated that the family Malagazziidae has no permanent rudimentary marginal bulbs. We hereafter distinguish the rudimentary marginal bulbs which can develop into tentacular bulbs from the permanent marginal warts. In conclusion, *Malagazzia* is characterised by marginal rudimentary bulbs with excretory papillae that are changeable to tentacular bulbs that bear the tentacles.

Medusae having the peculiar characteristics of Malagazziidae were collected from Japanese waters. The medusae were identified as *Malagazzia* owing to the presence of four radial canals, excretory papillae on each tentacular bulb, and four-lipped manubria. However, the medusae exhibited a peculiar character; several enigmatic brown spots like oil droplets are present on their gonads and manubriums. Although the number varied among the medusae, the structures always appeared in the adult medusae. This is a character unique to this genus. Considering additional characters by which we can distinguish this species from others in Malagazzia, we determined that the medusae represent an undescribed species and, thus, we describe it in this study. The new hydrozoan species described here appears to closely resemble a species of medusa that has been recorded in several field guides in Japan and exhibited in aquaria. These medusae have received a common name in Japan, but it is uncertain whether this name can corresponds to the new species we describe herein. We also briefly discuss the challenge of common names used in Japanese aquaria.

## Materials and methods

### Collection and observation of living medusae

**Figure 1.**
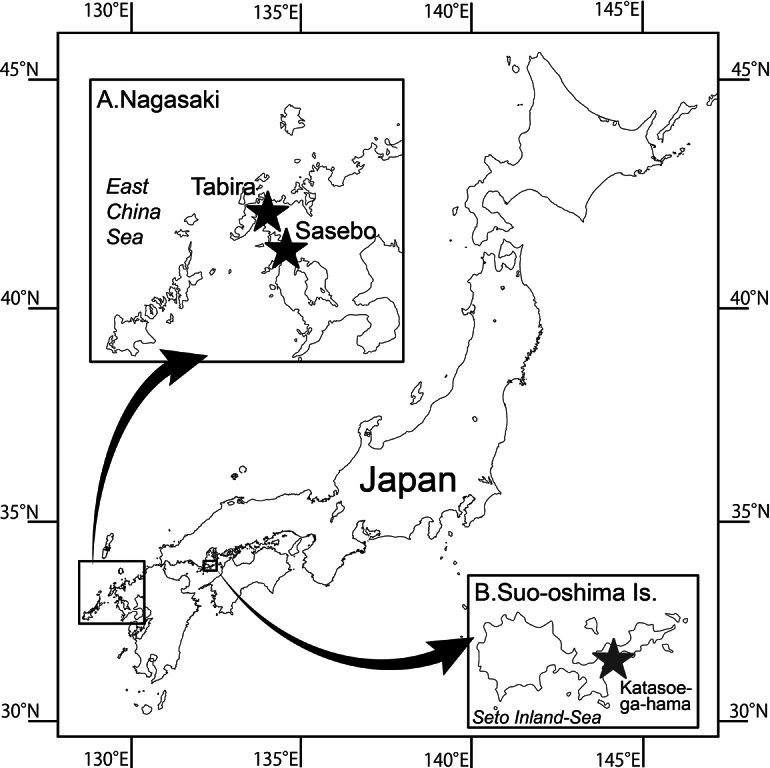
Sampling localities of *Malagazzia michelin*. **A**. Tabira and Sasebo, Nagasaki Pref. (indicated by black stars); **B**. Katasoe-ga-hama, Yamaguchi Pref. (by grey star).

Several medusae of *Malagazzia* specimens were collected from three localities (Fig. 1): Tawara-ga-ura, Sasebo, Nagasaki Prefecture, Tabira, Hirado, Nagasaki Prefecture, and Katasoe-ga-hama, Yashiro-Jima Island, Yamaguchi Prefecture; see material examined for the dates and collectors. The medusae were discovered directly by the naked eye from land, captured with a dipper or dip net (mesh size approximately 0.2 mm) with long handles, and put into bottles or buckets with sufficient sea water to observe them live. The collected medusae were transported to the Saikai National Park Kuju-kushima Aquarium and Tsuruoka City Kamo Aquarium. Some medusae were observed through binoculars and pictures were taken for morphological analyses. As specimens, samples were preserved in 5% formalin seawater solution (v/v) or 99% ethanol. The remaining medusae were kept in plankton-kreisels or beakers filled with seawater (at 20 °C at the Kuju-kushima and Kamo aquaria), fed on *Artemia* juveniles, and sometimes exhibited in the aquaria.

### Observation of life cycle

Development of polyps was observed in monoculture tanks where matured medusae were kept at the Kamo and Kuju-kushima aquaria. We also kept the polyps in glass bottles (at 20 °C at both aquaria), fed them with juvenile *Artemia*, and observed their morphology. Finally, a generation of juvenile medusae were observed. Thus, we were able to document their development by preserving a specimen on every alternate day.

In addition to the new species, we observed *Malagazzia hirsutissima*, determined according to Akiyama et al. (2013), for which we had succeeded in obtaining polyps and juvenile medusae at the Kuju-kushima Aquarium. We kept the *M. hirsutissima* polyps in glass bottles (at 20–23 °C), fed with juvenile *Artemia*, and observed their morphology. After the production of juvenile medusae, we also observed their primary development. Finally, we compared the morphology of *M. hirsutissima* with that of the new species described herein.

### Morphological observation

The living medusae, polyps, or preserved specimens were observed using stereo- or binocular microscopes (OLYMPUS MVX10, SZX16, or SZX17) and photographed by cameras mounted on the microscopes (OLYMPUS DP70, E-330, or BHC3-1080 [Beijing Bestscope Technology Co., Ltd]). Measurements were taken of umbrella size, tentacles, tentacle bulbs, peduncle and lips, radial canals, gonads, manubrium, and tentaculiform structures with reference to Bouillon et al. (2006).

Formalin-preserved specimens were deposited at **NSMT**, National Museum of Nature and Science, Tsukuba.

### Phylogenetic analysis

DNA was extracted from the medusae cultured in both aquaria. Medusae specimens were preserved in 99% EtOH and DNA was extracted using the ChargeSwitch gDNA Micro Tissue Kit (Invitrogen). PCR amplifications were performed for mitochondrial 16S rDNA following the primers and protocol by Miranda et al. (2016). The PCRs were performed in a 10-µL reaction volume, consisting of 0.4 µL of forward and reverse primers (25 µM), 2.0 µL of EmeraldAmp PCR Master Mix (TaKaRa Bio), and 3.4 µL of distilled water. The PCR products were processed with Exonuclease I and shrimp alkaline phosphate (Exo-SAP) before sequencing. Sequencing reactions were performed using PCR primers. Sequencing was conducted at Fasmac Co. Ltd (Kanagawa, Japan). Sequences of each marker were individually assembled by GeneStudio v. 2.2.0.0 (http://genestudio.com). New sequences obtained in this study have been deposited in GenBank (Table 1).

**Table 1.** Accession numbers of the data set used in phylogenetic analysis. Except *Malagazzia michelin* sp. nov., the data set was obtained from GenBank.

Higher taxa	Families	Genus	Species	Locality	16S
Leptothecata					
	Malagazziidae	**Malagazzia**	**michelin**	**Nagasaki1**	**LC911363**
		**Malagazzia**	**michelin**	**Nagasaki2**	**LC911364**
		**Malagazzia**	**michelin**	**Suo-Oshima**	**LC911365**
		* Malagazzia *	sp.	(China)	KF962491
		* Malagazzia *	carolinae	(Gulf of Mexico)	MN355840
		* Malagazzia *	carolinae	(Gulf of Mexico)	MN355841
		* Malagazzia *	carolinae	(Gulf of Mexico)	MN355842
		* Malagazzia *	condensum		JQ715906
		* Malagazzia *	condensum		JQ715907
		* Malagazzia *	condensum		JQ715908
		* Octophialucium *	aphrodite		MW528632
		* Octophialucium *	indicum		AY787897
		* Octophialucium *	irregularis		MW528655
		* Octophialucium *	medium		JQ715913
	Aequoreidae	* Aequorea *	aequorea		AY512518
		* Aequorea *	coerulescens		KT266599
		* Aequorea *	conica		HM053552
		* Aequorea *	victoria		EU305469
	Blackfordidae	* Blackfordia *	virginica		KT266605
	Lovenellidae	* Hydranthea *	margarica		DQ855932
	Eirenidae	* Eutima *	curva		FJ550514
	Campanulariidae	* Campanularia *	sp.		FN424118

For phylogenetic analyses, sequence data of some species of Leptothecata were obtained from GenBank with reference to Maronna et al. (2016). In that study, Malagazziidae were shown as being related to Aequoreidae; thus, we selected these sequences with reference to Maronna et al. (2016) and added members of Malagazziidae from GenBank. The datasets were aligned using MAFFT v. 7.402 (Katoh and Standley 2013) using the default settings. Ambiguously aligned regions were eliminated using Gblocks v. 0.91b (Castresana 2002) with sequence type as DNA and default parameters. Next, the files were processed in Kakusan 4 (Tanabe 2011) to concatenate and test substitution models for both RAxML and MrBayes analyses. Maximum-likelihood analyses were performed in RAxML-VI-HPC (Stamatakis 2006) with the GTR + Γ evolutionary model recommended by Kakusan 4 and evaluated using 100 bootstrap replicates. Bayesian inference (BI) was conducted using MrBayes v. 3.2.6 (Ronquist and Huelsenbeck 2003) with GTR + Γ, the recommended substitution parameter. The Markov Chain Monte Carlo runs were carried out as described by Izumi et al. (2023), except that the number of generations were 5,000,000 in the present analysis.

## Results

### Description

**Phylum Cnidaria Verrill, 1865**

**Class Hydrozoa Owen, 1843**

**Order Leptothecata Cornelius, 1992**

**Family Malagazziidae Bouillon, 1984**

**Genus *Malagazzia* Bouillon, 1984**

(New Japanese name: watage-kurage-zoku)

#### Malagazzia michelin

Taxon classificationAnimaliaLeptothecataMalagazziidae

Izumi et al. sp. nov.

D44EE5F5-15D3-50B6-A811-F52AA6ACEDBF

https://zoobank.org/706B18F1-FE2E-4941-907B-8782E7248908

**Figure 2.**
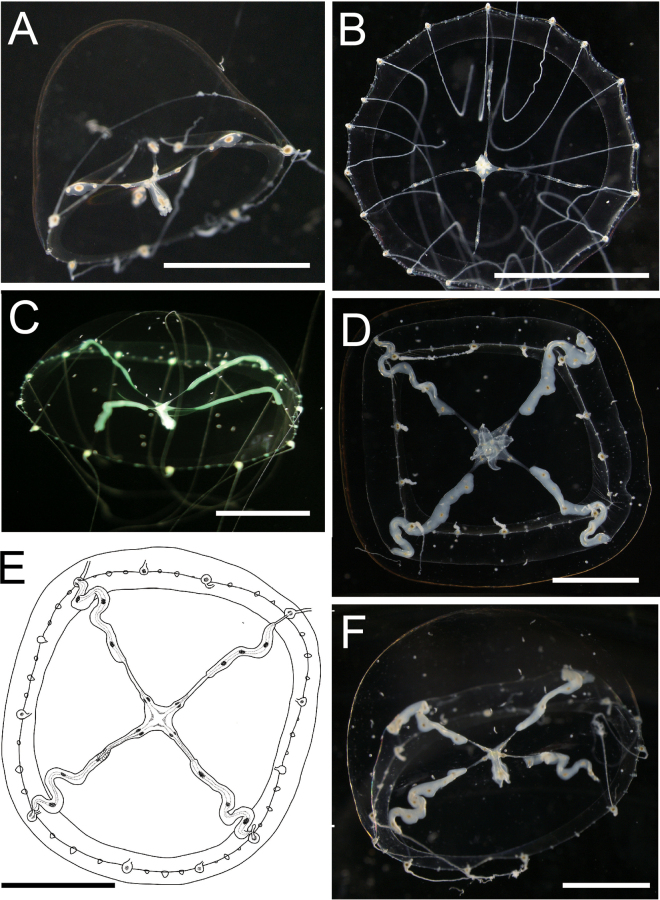
External view of the living medusae of *Malagazzia michelin*. **A, B**. NSMT-Co 1933; **A**. Lateral view; **B**. Oral view; **C**. Lateral view of Kuju-kushima 3 with lighting; **D–F**. Kuju-kushima 1; **D**. Aboral view; **E**. Sketch from aboral side; **F**. Lateral view. Scale bar: 5 mm.

**Figure 3.**
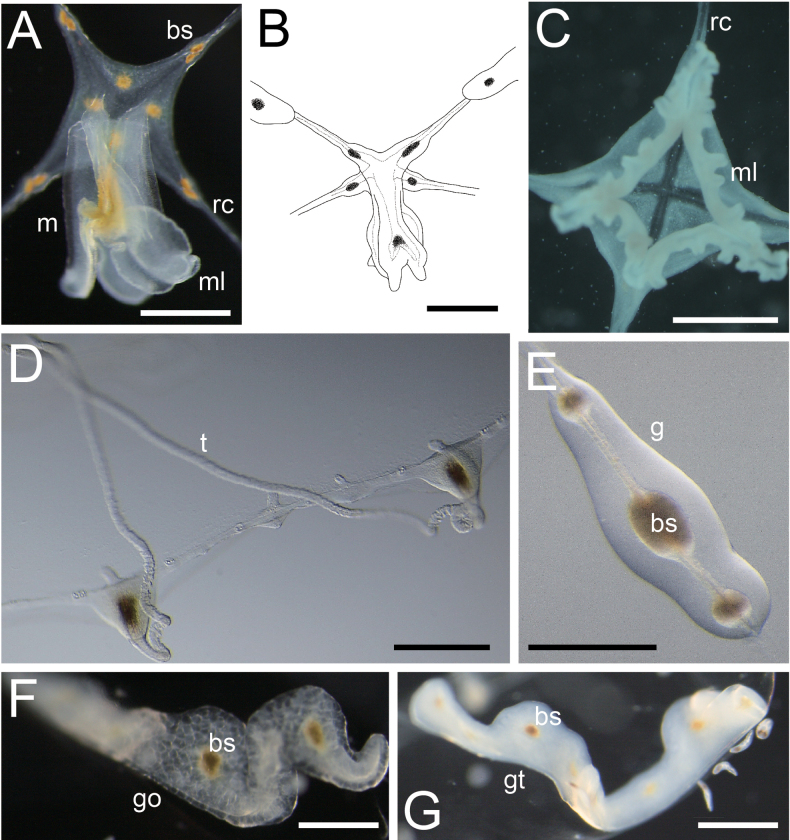
Enlarged view of the structures of living *Malagazzia michelin*. **A–C**. Manubrium, lips and radial canals; **A, B**. Closed lips (Kuju-kushima 1); **C**. Open lips (NSMT-Co 1933); **D**. Tentacles and tentacular bulbs (Kuju-kushima 1). **E–G**. Gonads; **E**. Immature (CMNH-ZG 1933); **F**. Mature female (Kuju-kushima 2); **G**. Mature male (Kuju-kushima 1). Abbreviations. bs: brown spot; g: gonad; go: gonad (ovary); gt: gonad (testis); m: manubrium; ml: mouth lip; rc: radial canal; t: tentacle. Scale bar: 0.1 mm

**Figure 4.**
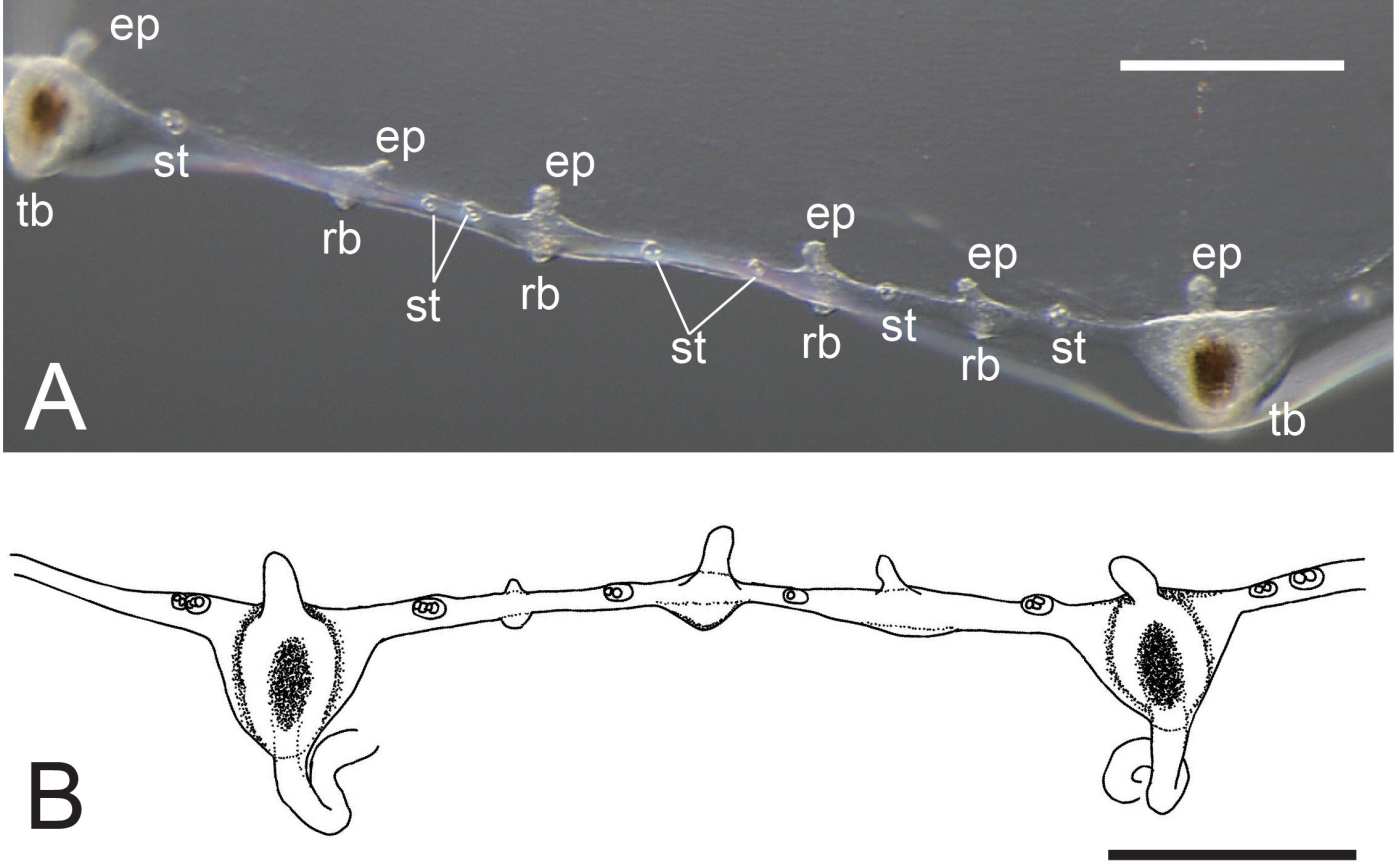
**A**. Enlarged view of the margin of umbrella; **B**. sketch of the same. Both NSMT-Co 1934. Abbreviations. ep: excretory papillae; rb: rudimentary marginal bulbs; st: statocyst; tb: tentacular bulb. Scale bar: 0.1 mm

**Figure 5.**
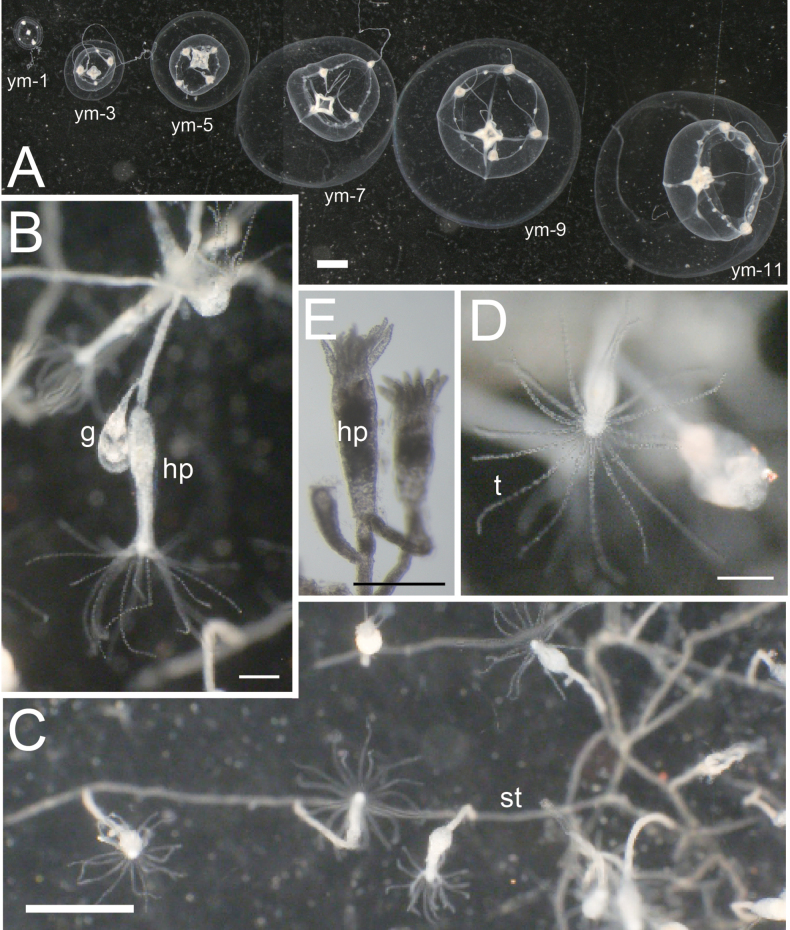
Polyps and juvenile–young medusae of *Malagazzia michelin*. **A**. Development of juvenile medusae (NSMT-Co 1936). From left side, day 1, 3, 5, 7, 9, 11 from release; **B–E**. Polyps (NSMT-Co 1937 or same colony); **B**. Enlarged lateral view of a hydrothecal pedicel and a gonotheca; **C**. Creeping hydrorhiza and several hydrothecal pedicels; **D**. Oral view of a hydrothecal pedicel; **E**. Specimen of hydrothecal pedicels and gonothecas (preserved). Abbreviations: g: gonothecata; hp: hydrothecal pedicel; st: stolon; t: tentacle; ym-x: young medusa (x days after released). Scale bar: 1 mm (thick); 200 µm (slender).

Figs 2, 3, 4, 5 New Japanese name: ama-no-gawa-kurage

##### Material examined.

***Holotype***: Japan – whole medusa specimen preserved in 5% (v/v) formalin seawater solution, male, umbrella diameter 17 mm; Yamaguchi Prefecture, Yashiro-Jima Island, Katasoe-ga-hama (Fig. 1B); 30 Sep. 2015; collected by Yoshimi Hamatsu and Hiroaki Uchida, preserved by Sho Toshino; collected on sea surface; **NMST-Co 1931**.

***Paratype* 1**: Japan – whole medusa specimen preserved in 5% (v/v) formalin seawater solution, male, umbrella diameter 9 mm; Yamaguchi Prefecture, Yashiro-Jima Island, Katasoe-ga-hama (Fig. 1B); 30 Sep. 2015; collected by Yoshimi Hamatsu and Hiroaki Uchida, preserved by Sho Toshino; collected on sea surface; **NMST-Co 1932. *Paratype* 2**: Japan – whole medusa specimen preserved in 5% (v/v) formalin seawater solution, male, umbrella diameter 10 mm; Nagasaki Prefecture, Sasebo City, Tawara-ga-Ura (Fig. 1A); 17 Aug. 2008; Hisashi Akiyama; collected on sea surface; **CMNH-ZG 1933. *Paratype* 3**: Japan – whole medusa specimen preserved in 5% (v/v) formalin seawater solution, female, umbrella diameter 10 mm; cultured at the Kuju-kushima Aquarium from the polyps; 25 Jan. 2012; Hisashi Akiyama; collected from the tank; **CMNH-ZG 1934. *Paratype* 4**: Japan – whole medusa specimen preserved in 5% (v/v) formalin seawater solution, juvenile, umbrella diameter 10 mm; cultured at the Kuju-kushima Aquarium from the polyps; 25 Jan. 2012; Hisashi Akiyama; collected from the tank; **NMST-Co 1935**.

##### Others.

**Voucher 1**: Japan – series of seven whole medusae specimens preserved in 5% formalin solution, juvenile, umbrella diameter 1–6 mm; cultured at the Kamo Aquarium, reproduced from the polyps; preserved on alternate days starting from polyp generation from 23 Sep. to 3 Oct. 2023; Shuhei Ikeda; collected from the tank; **NMST-Co 1936. Voucher 2**. Japan – Part of the polyp colony specimen preserved in 5% formalin solution; cultured at the Kamo Aquarium; 2 Oct. 2023; Shuhei Ikeda; collected from the tank; **NMST-Co 1937**.

##### Other materials

(only pictures remain; used for reproduction and observation of lifecycles). **Kuju-kushima-1**: Japan – whole medusa, male, umbrella diameter ca 20 mm; Nagasaki Prefecture, Hirado City, Tabira (Fig. 1A); 25 Jul. 2017; Yuichi Nozoe; collected on sea surface. **Kuju-kushima-2**: Japan – whole medusa, female, umbrella diameter ca 20 mm; Nagasaki Prefecture, Hirado City, Tabira (Fig. 1A); 25 Jul. 2017; Yuichi Nozoe; collected on sea surface; **Kuju-kushima-3**: Japan – whole medusa, male, no data of umbrella diameter; Nagasaki Prefecture, Hirado City, Tabira (Fig. 1A); 25 Aug. 2012; Shiori Horinouchi; collected on sea surface.

##### Diagnosis.

*Malagazzia michelin* sp. nov. is defined by the following characteristic features: hemispherical umbrella; manubrium with four long lips; without gastric peduncle; gonads on the middle part of radial canals (connected neither to lip or manubrium); four primary radial canals; tentacular bulbs more numerous (12–20); radial canals not branched; radial canals completely surrounded by gonads; brown spots on manubrium and gonads; number of tentacles between radial canals uneven; brown endodermal cores in tentacle bulbs; rudimentary bulbs and statocysts between tentacular bulbs; excretory papillae on each tentacular and rudimentary bulb.

##### Description.

***Medusae***. Umbrella diameter and height approximately 12–20 mm and 6–10 mm, respectively (holotype: 16 mm / 7 mm). Umbrella flat to hemispherical (Fig. 2A, B). Umbrella with mesoglea, noticeably thickened on apex (Fig. 2A). Exumbrella transparent (Fig. 2A, B, D, F), without nematocysts. Manubrium at the centre of umbrella, tubular, cruciform, colour translucent white and pale brown in centre (Fig. 3A). Gastric peduncle absent. Manubrium length approximately 1.0–2.5 mm, 1/8–1/10 of the length of the umbrella diameter, sometimes a little extended beyond umbrella margin. Mouth cruciform, with four long, frilled lips (Fig. 3B, C). Radial canals four, extending from the base of manubrium to edge of the umbrella (Figs 2, 3A–C), corresponding with the cruciform orientation of manubrium and mouth, and connected to tentacle bulbs on the marginal end (Fig. 2E). Radial canals narrow, straight to S-like winding, depend on shape of surrounding gonads. Gonads four, at centre of radial canals, approximately 3/4–4/5 length of radial canals, neither connected to manubrium nor tentacular bulbs (Fig. 2). Shape of gonad linear to S-like in shape, translucent or whitish in colour; translucent in immature phase (Fig. 3E) or matured ovary (Fig. 3F) and whitish in matured testis (Fig. 3G). Along base of four corners of the cruciform manubria to radial canals surrounded by gonads, presence of egg-like structures filled with dark brownish pigment: four on each side, one or two on manubrium (Fig. 3A), and one to five (generally two or three) on gonads (Figs 2, 3). Tentacles variable in number, between 12 and 20, simple, cord-like, approximately 3–4 times longer than umbrella height when elongated, covered with nematocysts, slightly translucent whitish (Fig. 3D), tightly coiled during constriction. Tentacle bulbs same number as tentacles, egg-shaped or triangular, on umbrella margin, having brown endodermal cores inside (Fig. 4), not located at equal intervals (Fig. 2), four tentacular bulbs corresponding with radial canals (Fig. 2). Rudimentary bulbs approximately 50–70, uneven in number, 2–4 between tentacles, no correspondence with radial canals (Fig. 4). Statocyst present, one or two between rudimentary bulbs, and 3–8 between tentacular bulbs. One to four (usually two) statoliths in each statocyst (Fig. 4). One excretory papilla on adaxial side on each tentacular and rudimentary bulb. Velum present, narrow, approximately 1/6–1/8 of umbrella diameter (Fig. 2B, F).

***Polyps*** (Fig. 5B–E): colonies stolonal; arising from creeping hydrorhiza. Stolons long, slender, smooth, giving rise to short pedicels. Hydrothecal pedicels usually smooth, slender, approximately 0.7–1.1 mm in length and 0.1–0.15 mm in width, with a conical operculum formed by numerous convergent segments; tentacles arising from hydranth, approximately 16, slender; gonotheca ovate, approximately 0.4 mm in length and 0.2 mm in width, with flat to rounded distal end, gradually tapering towards proximal end, arising from base of hydrothecal pedicels.

***Development of juvenile medusae*** (NSMT-Co 1936; Fig. 5A).

**Day 1**: umbrella diameter and height of just-released medusa approximately 1 mm. Mesoglea approximately 1/8 as thick as umbrella height. No gastric peduncle and gonads. Manubrium small, cylindrical, length 1/3–1/2 of umbrella height. Mouth without lips. Radial canals four, already connecting both to manubrium and margin of umbrella. Tentacle bulbs four; two noticeably smaller than the other two, and alternately larger and smaller. Tentacles present only on larger bulbs. One undeveloped rudimentary bulbs and two statocysts between each tentacular bulb. **Day 3**: manubrium becoming cruciform. Number of rudimentary bulbs increasing by two between each tentacular bulb. **Day 5**: two tentacles growing from two small tentacular bulbs. Small lips growing on mouth. **Day 7**: four tentacular bulbs becoming almost same in size. Primary gonads appearing on radial canals. **Day 9**: additional tentacular bulbs appearing between the first four bulbs. Lips noticeably separating into four. **Day 11**: immature gonads surrounding every radial canal. Manubrium and mouth with four lips becoming almost same in shape as that of adult. Eleven tentacular bulbs and 16 rudimentary bulbs finally developing.

During rearing of the species at the Kamo and Kuju-kushima aquaria, the maximum size of *M. michelin* sp. nov. was approximately 10 mm in diameter, never developing to the size of medusae obtained from the wild sea. It took approximately 1–2 months at 20 °C to reach this maximum size.

##### Etymology.

Michelin is derived from the “Michelin Guide,” the famous restaurant guidebook. Brown spots are present on the manubrium base or gonads of this species. Especially in the gonads, the number of spots is not fixed and increases with development. These spots can be associated with the “stars” of the Michelin guide, bestowed depending on the quality of the restaurant; therefore, we named the species after the shortened name of the guidebook. ***Derivation of Japanese name***: the gonads of these medusae, patches within white winding strips, are associated with the Milky Way with several stars in the night sky.

##### Phylogenetics

**Figure 6.**
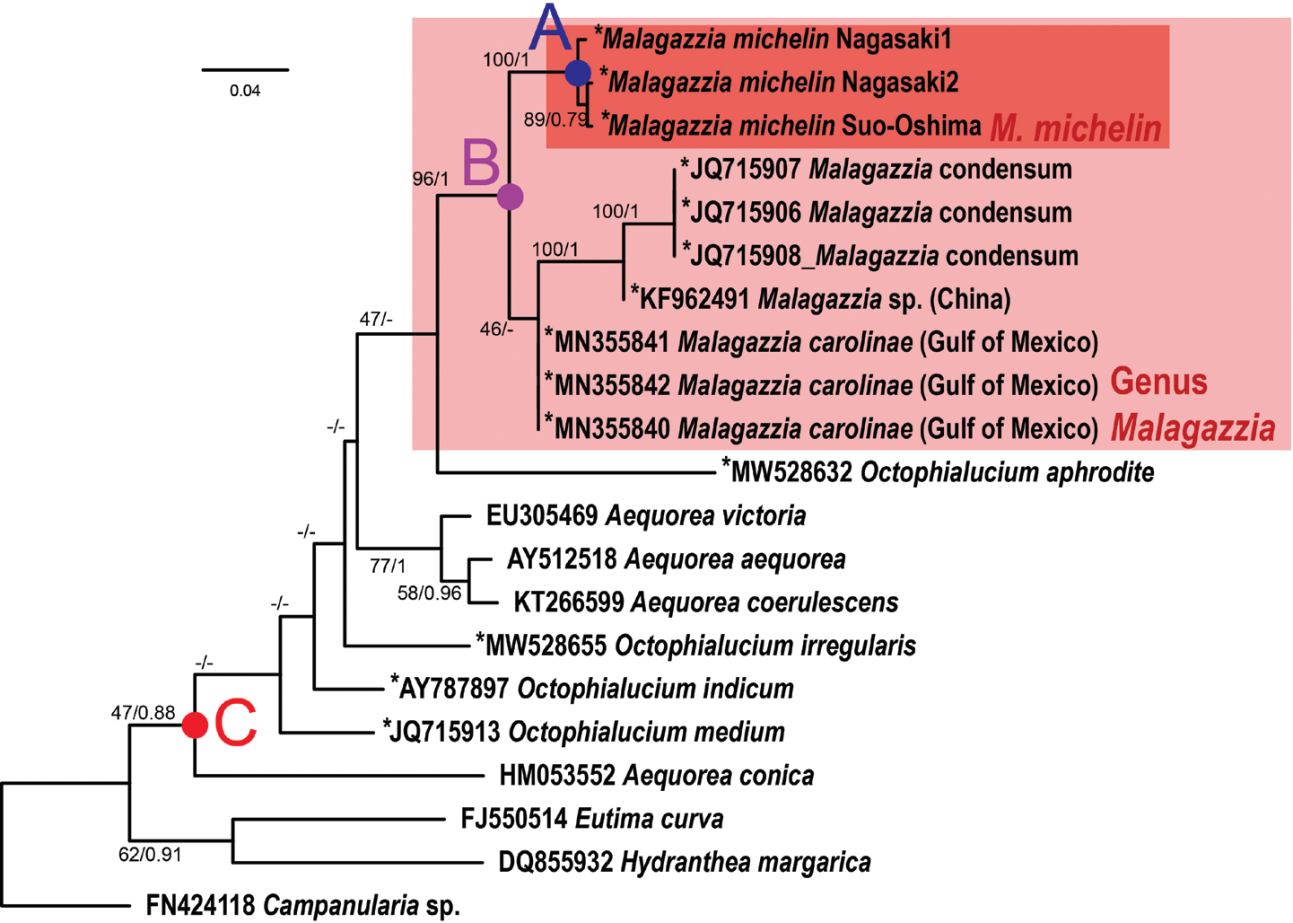
Phylogeny of *Malagazzia michelin* in Leptothecata. Dark red box indicates the clade of *M. michelin* and light one does the clade of genus *Malagazzia*. The numbers of node indicate bootstrap values (BV) of ML method followed by posterior probabilities (PP) of Bayes method; only described when the node was supported in BV > 40 or PP > 0.75.

(Fig. 6). Results of the phylogenetic analysis of Malagazziidae and Aequoreidae show that *Malagazzia michelin* sp. nov. was most closely related to *M. condensum* and *M. carolinae* (node B; bootstrap value [BV]/posterior probability [PP] = 96/1); thus, *Malagazzia* species were recovered as monophyletic. *Malagazzia michelin* from two localities were nested with high reliability (node A; BV/PP = 100/1). At the family level, Malagazziidae (OTU indicated by *) was found to be paraphyletic. Although the species belonging to Malagazziidae and Aequoreidae were monophyletic (node C; BV/PP = 47/0.88, a slightly low value), *Octophialucium*, a member of Malagazziidae, was positioned in several parts of the tree and support values of the phylogenetic tree was too low in several nodes (lower than 50 in bootstrap values and often polytomous in the Bayesian tree).

#### Malagazzia hirsutissima

Taxon classificationAnimaliaLeptothecataMalagazziidae

Akiyama, Horinouchi, & Kubota, 2013

44FC6450-6436-5056-8E49-225A43B4DC1C

**Figure 7.**
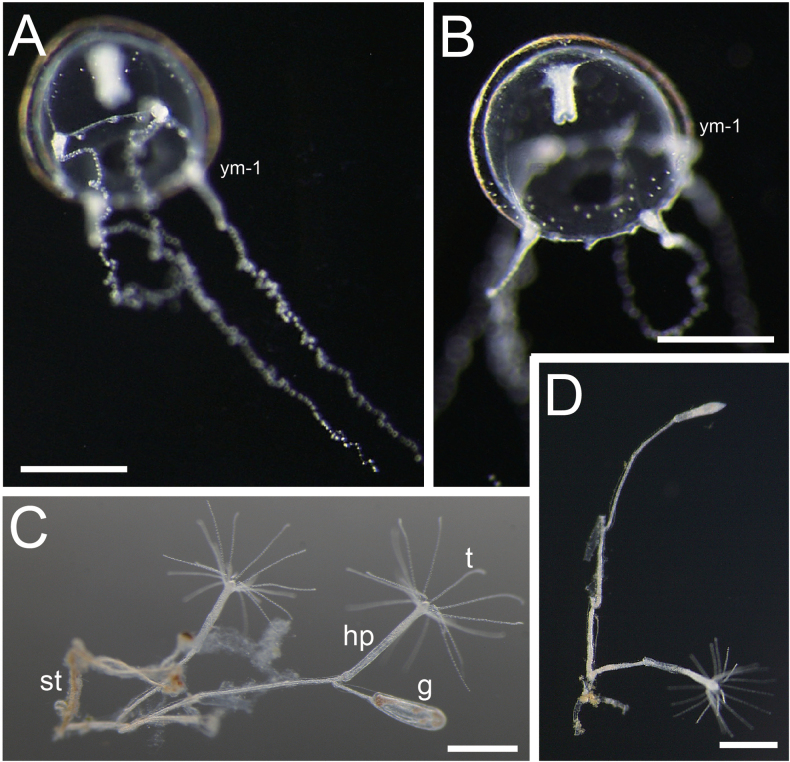
Polyps and juvenile medusa of *Malagazzia hirsutissima*. **A, B**. Juvenile medusae (NSMT-Co 1938); **C, D**. Polyps (NSMT-Co 1939 or same colony); **C**. Hydrothecal pedicels and a gonothecas; **D**. Hydrorhiza and two hydrothecal pedicels. Abbreviations: g: gonothecata; hp: hydrothecal pedicel; st: stolon; t: tentacle; ym-x: young medusa (x means the days after released). Scale bar: 0.5 mm.

Fig. 7 Common Japanese name: watage-kurage

##### Material examined.

**Voucher 1**: Japan – two whole medusae specimens preserved in 5% formalin solution, juvenile, umbrella diameter 1.0–1.4 mm; cultured at the Kuju-kushima Aquarium reproduced from the polyps; preserved on alternate days starting from polyp generation from 11 Sep. 2024; Hisashi Akiyama; collected from the tank; **NMST-Co 1938. Voucher 2**: Japan – part of the polyp colony preserved in 5% formalin solution; cultured at the Kuju-kushima Aquarium; 11 Sep. 2024; Hisashi Akiyama; collected from the tank; **NMST-Co 1939**.

##### Description.

***Adult medusa***: see Akiyama et al. (2013).

***>Polyps*** (NSMT-Co 1939; Fig. 7C, D): polyp shape almost resembling that of *Malagazzia michelin*. Colonies stolonal; arising from creeping hydrorhiza. Stolons long, slender, smooth, giving rise to short pedicels. Hydrothecal pedicels usually smooth, slender, approximately 1.0–1.4 mm in length and 0.7–1.0 mm in width, with a conical operculum formed by numerous convergent segments; tentacles arising from hydranth approximately 16–20, slender; gonotheca shaped like a rice-grain, approximately 0.6 mm in length and 0.2 mm in width, with rounded distal end, gradually tapering towards proximal end, arising from stolon or base of hydrothecal pedicels.

***Development of juvenile medusae*** (NSMT-Co 1938; Fig. 7A, B): **Day 1**: umbrella diameter and height of just-released medusa approximately 0.7–0.9 mm. Mesoglea approximately 1/8 as thick as umbrella height. No gastric peduncle and gonads. Manubrium small, cylindrical, approximately 1/2 in length as umbrella height. Mouth with small lips. Radial canals four, already connecting both to manubrium and umbrella margin. Tentacle bulbs four, same size. Tentacle on each of four bulbs, slender, 2–3 mm in length, with scattered nematocyst-like particles. Small, wart-like structures (primary rudimentary bulbs potentially developing into tentacular bulbs) present between each bulb, and eight statocysts on margin. One statolith in each statocyst. Scattered nematocyst-like particles on umbrella surface.

During rearing at the Kuju-kushima Aquarium, the maximum developmental size of *M. michelin* was 13.6–16.6 mm in diameter. Different from *M. michelin*, this species developed nearly to the size of medusae obtained from the wild sea. It took approximately 3 months at 20 °C to reach this maximum size.

## Discussion

### Taxonomic remarks for *M. michelin* sp. nov.

Our study places *M. michelin* in the genus *Malagazzia* as defined by Bouillon (1984). As mentioned in the Introduction, the most characteristic feature of this genus is the excretory papillae on the tentacular and warts-like rudimentary bulbs. These features were distinct in *M. michelin*; hence, there is no doubt regarding the classification of *M. michelin* at the genus level.

The comparison of *M. michelin* with the other *Malagazzia* species is shown in Table 2 with the addition of the present study to Akiyama et al. (2013). There have been eight nominal species are present in *Malagazzia* (Akiyama et al. 2013; Schuchert 2024). When distinguishing *M. michelin* from other malagazzids, the most characteristic features were the brownish spots on the gonads and manubrium. These spots did not look like simple concentration of pigments, but rather as structures surrounded by membrane-like thin skin. Although the role of these structures is unknown, these brownish spots can be the most useful key by which we can distinguish *M. michelin* from other malagazzids. Only *Malagazzia multitentaculatum* (Menon, 1932) is known to possess structures like pigment spots, although there no mention of the pigment spots was made in the original description (Menon 1932), Bouillon (1984) described the four lines of intermediary pigmented spots extending over the ceiling and lateral walls of the manubrium. This description is similar to the morphology of *M. michelin*, although the spots never form lines. However, both Menon (1932) and Bouillon (1984), and others, such as Kramp (1961, 1968), make no mention of spot-like structures on gonads which mostly characterize *M. michelin*. These structures do not look like simple concentration of pigment but instead appear like an oil droplet surrounded by thin skin (compare Fig. 3A, E), which is not known in any other described malagazzian medusae. In addition, although *M. michelin* is larger than *M. multitentaculatum* in maximum diameter, the number of tentacles and statocysts never exceed those of *M. multitentaculatum* (Table 2). Interestingly, according to Bouillon et al. (2004) and Wang et al. (2018), Malagazziidae medusae do not have a permanent rudimentary marginal bulb. Thus, it is unnatural to identify *M. michelin* as *M. multitentaculatum* because even if *M. multitentaculatum* grows to the size of *M. michelin*, the number of tentacles will be far more than in *M. michelin*.

**Table 2.** Comparisons among *Malagazzia* including the present new species.

Species	Max. bell diameter (mm)	Radial canals		Gonads			Shape of manubrium	Shape of lips	Gastric peduncle	Number of tentacles
		Shape	Number	Shape	Position	Position in young medusae				
***Malagazzia michelin* sp. nov**.	**Up to 20**	**Narrow and twisted**	**4**	**Linear to S— shaped egg—like brown stractures**	**Almost 1/1 never connect to tentacular bulbs**	**Almost 1/1 or slightly proximal**	**Tubular, cruciform, brown spots**	**Four, long frilled**	**Absent**	**12–20**
*M. carolinae* (Mayer, 1900)	14	Narrow and straight	4	Linear	Distal 1/4	—	Flask—shaped	Four, simple, curved	Absent	16
*M. multitentaculatum* (Menon, 1932)	Up to 14	Narrow and straight or twisted	4	ribbon—like, twisted	Almost 1/1 Sometimes nearby tentacular bulbs	Distal	Pitcher—shaped, pigment spots	Four, long fimbricated	Absent	25**–**32
*M. condensum* (Kramp, 1953)	5–7	Very narrow	4	Band—shaped	Proximal 1/4–1/3	—	Quadrangular, short	Simple	Absent	About 12
*M. taeniogonia* (Chow & Huang, 1958)	Up to 15.3	Narrow and straight	4	Slightly twisted wide ribbons	Distal	—	Short, quadrate	Frilled	Absent	8
*M. curviductum* (Xu & Zhang, 1978)	4–10	S—shaped	4	S—shaped	Almost 1/1	—	Short	—	Absent	12–16
*M. cyphogonia* (He & Xu, 1982)	10–14	Narrow and straight	4	Band—shaped, twisted as curve	Distal	—	Quadrate, short	Large, simple	Absent	14**–**19
*M. monocanalis* (Xu, Hung & Liu, 2006)	10	Straight	2	Ovoid	Distal	—	Cylinder—like, short	Simple	Absent	15
*M. hirsutissima* Akiyama, Horinouchi & Kubota, 2013	8.35**—**14.20	Narrow and straight	4	Linear	Middle 2/3	—	Short, slender	Crenulated and folded many times	Absent	112**–**218
Species	Number of rudimentary bulbs		Number of statocysts between two adjacent tentacles (total number of statocysts)	Number of statoliths per statocyst	Color	Polyp		Geographical distribution	Source	
	total	between two adjacent tentacles				Shape	Position of gonotheca			
***Malagazzia michelin* sp. nov**.	**50–70**	2–4	25**–**34	1–4	**Tentacle bulbes and gonads translucent to pale white Brownish spots in them**	**Stolonal, creeping**	**Base of hydrothecal pedicle**	**Seto Inland-Sea and Northwesten region of Kyusyu, Japan**	**Present study**	
*M. carolinae* (Mayer, 1900)	48	—	4 (64)	2	Tentacle bulbes, proboscis, gonads bright yellow**—**green	N/A	N/A	Charleston Harbor,Carolina; Tortugas, Florida; Palao Islands; Great Barrier Reef, Australia; Chefoo, China	Mayer (1900, 1910) Uchida (1947)Kramp (1953)Chow and Huang (1958)	
*M. multitentaculatum* (Menon, 1932)	—	3–4	— (more than 150)	Usually 2 (2**—**4)	Tentacle bulbs brown, gonads light brownish yellow	N/A	N/A	Madras, India	Menon (1932) Bouillon (1984)	
*M. condensum* (Kramp, 1953)	—	1–3	3–4	—	—	Stolonal, creeping	Directly from stolon	Great Barrier Reef South China Sea	Kramp (1953) Bouillon (1984) Wang et al. (2018)	
*M. taeniogonia* (Chow & Huang, 1958)	40–64	5–8	4–8	—	Manubrium, stomach and tentacle bulbs green, gonads light red**—**green	N/A	N/A	Chefoo, China	Chow and Huang (1958)	
*M. curviductum* (Xu & Zhang, 1978)	—	3	2–4	2	—	N/A	N/A	Guangdong and Fujian, China	Xu and Zhang (1978)	
*M. cyphogonia* (He & Xu, 1982)	—	2–4	1–4	—	Gonads brownish red	N/A	N/A	Yantai, China	He and Xu (1982)	
*M. monocanalis* (Xu, Hung & Liu, 2006)	15–30	1–2	2–4 usually 3	2	—	N/A	N/A	Changjiang River Estuary, China	Xu et al. (2006)	
*M. hirsutissima* Akiyama, Horinouchi & Kubota, 2013	Few	0, rarely 1 (0 or 1)	0**–**1 (55–174)	Usually 2 rarely 1, 3, 4	Manubrium, stomach, tentacle bulbs and gonads white	Stolonal, creeping	Base of hydrothecal pedicle	Northwesten region of Kyusyu, Japan	Akiyama et al. (2013)	

Concerning the distribution of these species, *M. multitentaculatum* is mainly recorded from the Indian Ocean and has never been recorded in the South China Sea although several *Malagazzia* species have been collected there (Wang et al. 2018). Therefore, the distribution of these two species are disjunct.

In addition, *M. michelin* can be distinguished from the other species by the following features: *M. hirsutissima* Akiyama, Horinouchi & Kubota, 2013 has far more noticeable tentacles; *M. monocanalis* Xu, Huang & Liu, 2007 can be distinguished by having only two radial canals; *M. carolinae* (Mayer, 1900) has linear gonads and radial canals, which differs from those of *M. michelin* in their shape; *M. taeniogonia* (Chow & Huang, 1958), *M. condensum* (Kramp, 1953), and *M. cyphogonia* (He & Xu, 1982) are distinguished from *M. michelin* in the distribution of gonads reaching the marginal side; statocysts of *M. curviductum* (Xu & Zhang, 1978) contain two statoliths in contrast to those of *M. michelin* in which statocysts contain 1–4 statoliths (Table 2).

In addition, a primary taxonomic key of *Malagazzia medusae* is presented here.

### Primary taxonomic key of *Malagazzia medusae*

Sources: Mayer 1900; Menon 1932; Uchida 1947; Kramp 1953; Chow and Huang 1958; Xu and Zhang 1978; He and Xu 1982; Bouillon 1984; Xu et al. 2006; Akiyama et al. 2013.

**Table:** 

A1	Radial canals 2	***M. monocanalis***
A2	Radial canals 4	**B**
B1	Radial canals and gonads almost straight	**C**
C1	Tentacles 16	***M. carolinae***
C2	Tentacles over 100	***M. hirsutissima***
B2	Radial canals and gonads twisting	**D**
D1	Gonad distributed on marginal side of radial canals	**E**
E1	Tentacles 8	***M. taeniogonia***
E2	Tentacles 14–19	***M. cyphogonia***
E3	Tentacles 20–25	***M. condensum***
D2	Gonads existing at the centre of radial canals	**F**
F1	Statoliths number 2 in every statocyst	***M. curviductum***
F2	Statolith number varies per statocysts	**G**
G1	Statocysts > 150. Manubrium pitcher-like	***M. multitentaculatum***
G2	Statocysts < 100. Manubrium cruciform	***M. michelin* sp. nov**.

Comparing the polyps of *M. michelin* with *M. hirsutissima* and the description of *M. condensum* by Bouillon (1984), the outline of whole polyp is not so different among these malagazzids, the position of gonophores apparently differs between them: gonotheca of *M. michelin* and *M. hirsutissima* arise from near the base of hydrantheca, but those of *M. condensum*, which has been the only species whose polyp was known, bear directly from the stolon (Bouillon 1984: fig. 23). However, it is a little premature to use the polyp morphology for the diagnostic key to distinguish species in *Malagazzia* because polyps are unknown in six of the nine species in this genus, including *M. multitentaculatum*, which most resembles *M. michelin*.

### Phylogenetic position of Malagazziidae

As described in the Introduction, three genera are present in Malagazziidae. Among them, DNA sequences of species in two genera, two species of *Malagazzia*, and four species of *Octophialucium* were obtained (Table 1). Although these two genera were related by having excretory papillae on both bulbs, several different features were present between them; for example, the number of radial canals and gonads in the medusa, which is one of the most characteristic features of hydrozoan taxonomy, differs between the two. Malagazzids have four radial canals and gonads (except for *M. monocanalis* in which only two canals exist; Xu et al. 2006), whereas medusae of *Octophialucium* have eight (Bouillon 1984; Bouillon et al. 2006).

Based on our phylogenetic analysis, *Malagazzia michelin* and the genus *Malagazzia* is confirmed as monophyletic given the present sampling (node A and node B of Fig. 6; BV/PP = 100/1 and 96/1, respectively). Though sampling of additional species of *Malagazzia* is necessary to further assess, currently this genus should be phylogenetically valid because of their monophyly; note that *M. carolinae* from Gulf of Mexico and *Malagazzia* sp. from China (deposited as *M. carolinae* in GenBank) are suggested to be paraphyletic. However, the status of family Malagazziidae is becoming questionable because *Octophialucium* is shown to be paraphyletic (Fig. 6). Although the most basal node (C; BV/PP = 47/0.88) contains all species of Malagazziidae, the clade also contains *Aequorea* species, family Aequoreidae Eschscholtz, 1829. Moreover, the reliability of several inner nodes (between node B and C in Fig. 6) were extremely low (BV ranges are far < 50 and several topologies were not supported in the Bayesian analysis). As the phylogenetic position of *Aequorea* and *Octophialucium* appear in several different locations, 16S RNA is limited in its ability to show genera and family nodes in the phylogenetic tree in this group. Therefore, we cannot discuss the phylogenetic arrangement of these families without more data.

### The importance and risk of common names in Japanese aquaria

*Malagazzia michelin* has been previously exhibited as “tsubuiri-sujiko-yawara-kurage” (meaning “salmon-roe laodicean jellyfish” in Japanese) at Kamo Aquarium. This common name was originally designated in a field guide by Kubota (2014). A species of Leptothecata resembling *M. michelin* has been collected in Japan; Kubota described the medusae from Nansei Island and Wakayama Pref. in a field guidebook (Kubota 2014), and some picture guides (e.g. Murai 2019) followed this description. However, applying this common name to *M. michelin* directly is problematic because these Japanese medusae have been identified as the species of *Laodicea* Lesson, 1843 at least in these field guides. Since it is confirmed that *Laodicea* in Laodiceidae Agassiz, 1862 and Malagazziidae are not so closely related to each other (Maronna et al. 2016), it is possible that the medusae species called “tsubuiri-sujiko-yawara-kurage” is not the same species as *M. michelin*; the could be visually similar but phylogenetically distant species.

In Japan, especially the ornamental animals like jellyfishes tend to be assigned common names first and foremost, and the scientific name corresponds temporarily to them without detailed inspection. Therefore, sometimes the scientific name turns out to be wrong and is re-named. For example, “giyaman-kurage”, one of the most famous hydrozoan jellyfish in Japanese aquaria identified as *Tima* Eschscholtz, 1829, had been exhibited as *Tima formosa* L. Agassiz, 1862 for several decades. Recently, however, this Japanese *Tima* species was distinguished from *T. formosa*, the Atlantic species, and described as a new species *Tima nigroannulata* (Calder et al. 2021). Now, *T. nigroannulata* is applied to the jellyfish known by the common name “giyaman-kurage”. In summary, since the use of common names cannot be avoided when exhibiting in aquaria for Japanese visitors, researchers need to determine the accurate species name for species to ensure correct classifications.

## Supplementary Material

XML Treatment for Malagazzia michelin

XML Treatment for Malagazzia hirsutissima

## Additional information

### Use of AI

No use of AI was reported.

### Data availability

All of the data that support the findings of this study are available in the main text.
